# Use of Non-Assigned Smoking Cessation Programs Among Participants of a Web-Based Randomized Controlled Trial

**DOI:** 10.2196/jmir.1172

**Published:** 2009-06-25

**Authors:** Brian G Danaher, Edward Lichtenstein, H Garth McKay, John R Seeley

**Affiliations:** ^1^Oregon Research InstituteEugeneORUSA

**Keywords:** Non-assigned treatments, treatment integrity, treatment fidelity, Web-based interventions, Internet, tobacco cessation

## Abstract

**Background:**

Concurrent with their enrollment in Web-based Randomized Controlled Trials (RCTs), participants can easily choose to use treatment programs that are not assigned in the study. The prevalence of using non-assigned treatments is largely unknown although it is likely to be related to the extent to which non-assigned treatments are: (a) easy to find and use, (b) low in cost, (c) well publicized, and (d) available from trusted sources. The impact of using other programs—both beneficial and detrimental—warrants additional research investigation.

**Objective:**

The aim of this report is to explore the extent to which participants enrolled in a Web-based intervention for smoking cessation used treatment methods that were not explicitly assigned (“non-assigned treatment”). In addition to describing the relation between using non-assigned treatments and smoking cessation outcomes, we also explore the broader issue of non-assigned program use by RCT participants in Web-based behavioral interventions, generally.

**Methods:**

We describe the use of other programs (as measured by self-report at the 3-month follow-up assessment) by 1028 participants who were randomized to the Web-based SHIP (Smokers’ Health Improvement Program) RCT which compared the Quit Smoking Network (QSN) treatment program and the Active Lives control condition. We examine the extent to which pharmacotherapy products were used by participants in the QSN condition (which explicitly recommended their use) and the Active Lives condition (which purposefully omitted mention of the use of pharmacotherapy). We also test for any between-condition impact of using non-assigned treatments and pharmacotherapy products on smoking cessation outcomes.

**Results:**

A total of 24.1% (248/1028) participants reported using one or more smoking cessation treatment programs that were not explicitly recommended or assigned in their treatment protocol. Types of non-assigned treatments used in this manner included individual counseling (1.7%), group counseling (2.3%), hypnotherapy/acupuncture (4.5%), pamphlets/books (12.6%), and other Web-based smoking cessation programs (9.0%). Participants who used non-assigned treatments were more likely to be female and have at least a high school education. Use of non-assigned Web programs was related to greater levels of self-reported smoking cessation measured at the 3-month assessment (OR = 2.63, CI = 1.67 - 4.14, *P* < .001) as well as the combined 3- and 6-month assessments (OR = 2.09, CI = 1.11 - 3.91, *P* = .022). In terms of reported medication use, there were no differences between conditions in the number of pharmacotherapy products used. However, more participants in the QSN condition used at least one pharmacotherapy product: 50.0% (262/524) vs 43.8% (221/504); χ^2 ^(1, N = 1028) = 3.90, *P* = .048. The use of pharmacotherapy and non-assigned treatment types showed a small but marginally significant correlation: *r*_1028_ = .061, *P* = .05.

**Conclusions:**

A noteworthy proportion of individuals recruited via the Internet to participate in a Web-based intervention used treatment programs and tools not formally assigned as a part of their research protocol. We consider factors likely to influence using non-assigned treatments and suggest ways that future research can begin to study more fully this important phenomenon which is likely to be found in any type of research, but may be particularly pronounced in minimal contact, Web-based intervention trials.

## Introduction

Research interest in Web-based health behavior change interventions is growing rapidly [1-3]. The power and convenience of current Internet search engines make it likely that online recruitment will reach many prospective participants for randomized controlled trials (RCTs). Consider, for example, data from NCI’s Health Information National Trends Survey [4] indicating that 58.4% of respondents looked for personally relevant health or medical information on the Internet. Similarly, the Pew Internet & American Life Project reported that 9% of all Internet users searched for quit-smoking advice [5]. The same computer skills that enable Web users to reach online health behavior change programs can easily be used by study participants to find—and use—other treatment programs concurrent with being enrolled in an RCT. In some cases, Web interventions encourage participants to explore the use of additional treatment resources [6].

In many other instances, researchers have not acknowledged or even reported upon the prevalence and impact of RCT participant use of non-assigned treatments in this manner. Literature germane to this topic includes treatment debriefing (eg, [7]), treatment integrity and fidelity (eg, [8]), and quality assurance of clinical trials (eg, [9]). In this paper we describe the extent to which participants enrolled in the Smokers’ Health Improvement Program (SHIP) project—a Web-based smoking cessation trial—reported that they used various treatment methods that had not been explicitly included in their assigned protocol.

## Methods

### The SHIP RCT

The SHIP smoking cessation RCT used online recruitment methods (ie, ad placement on Google and Yahoo search engines and links to affiliated sites) to enroll 2318 smokers from the US and Canada to participate in a randomized controlled trial. The trial was not registered, because enrollment started in spring 2005, before trial registration became mandatory. Prospective participants visited the recruitment website where they completed an online screening survey that included the 8-item Physical Activity Readiness Questionnaire (PAR-Q) [10]. Prospective participants had to be current smokers, at least 18 years of age, interested in quitting within the next 30 days, willing to engage in moderate physical activity, and have access to the Internet. Exclusion criteria included any positive answers on the PAR-Q used to identify individuals for whom physical activity might be inappropriate or individuals who should have medical advice concerning the type of activity most suitable for them. A more complete description of recruitment procedures and eligibility criteria has been reported in our outcome results paper [11].

Smokers who completed the screening and consent stages were randomized using a computer-based vector method to one of two Web-based programs: (a) the Quit Smoking Network (QSN) condition (N = 1159) or (b) the Active Lives control condition (N = 1159). Baseline data of 2318 study participants showed that most were women (70.5%), White (86.6%), urban (80.3%), married (61.6%), had at least some college education (68.2%), and smoked 1 - 2 packs of cigarettes each day (78.5%).

#### The QSN Intervention Condition

When study participants first used the Web-based QSN program, they were required to move through a series of Web pages that introduced key concepts and strategies of a combined behavioral-pharmacologic program for quitting smoking. Thereafter—and during subsequent visits—participants were free to choose any of a broad array of additional content on quitting and maintaining nonsmoking. The behavioral intervention was based on Social Cognitive Theory [12,13], and it provided modules (each having multiple Web pages) focused on getting ready to quit, developing a personal quitting plan, setting a personal quit date, avoiding and altering trigger situations, using substitutes, managing thoughts, and using strategies to manage mood. Tailored recommendations were provided to participants based on their baseline characteristics, and online videos of ex-smokers and a program expert were used on many Web pages to reinforce and model the use of program content and recommendations. The QSN program also provided access to a peer-to-peer Web forum, a moderated “Ask an Expert” forum, and an extensive library of additional content. Because participants were required to log in to the website using their unique usernames and passwords, it was possible to tailor portions of the program content to each participant’s smoking/nonsmoking status (checked at the start of each session) and to display online prompts recommending the review of program content that a participant had not yet explored.

The QSN program strongly advocated the use of pharmacological adjuncts and it contained a number of Web pages devoted to the use of Nicotine Replacement Therapy (NRT) and Zyban^®^. These Web pages provided an explanation of how to use these products, photos of representative products, supportive videos of smokers, interactive questions designed to elicit participant commitment to use these products, and agreement to see a doctor in order to obtain a prescription. NRT products included nicotine gum, patch, lozenge, spray, and inhaler.

#### The Active Lives Control Condition

The Web-based Active Lives control condition was a content-rich, multiple-module Web-based program that encouraged smokers to develop a personal physical activity program in order to become more fit which, in turn, would help them to quit smoking. The program guided each participant through a multi-step plan that included a motivational component (exploration of the benefits of physical activity and a clarification of personal goals and barriers), a behavioral action plan with extensive tracking features (eg, weekly activity schedules personalized to each participant’s schedule and types of activities), additional online resources (articles and “tip” sheets), and access to a Web Forum for peer support (distinct from the aforementioned peer forum in the QSN program). In contrast to the QSN condition, the Active Lives control condition purposefully omitted any reference to the use of pharmacotherapy (NRT or Zyban^®^).

#### Recommendations Regarding Use of Non-assigned Treatment/Resources for Smoking Cessation

Both the QSN and Active Lives programs encouraged participants to use the smoking cessation approaches featured in each website. However, participants were not explicitly cautioned against using other treatment programs or resources during and/or following their involvement with this study.

### Measures

#### Assessments

Assessment data were collected at screening, baseline, and at 3- and 6-month follow-up assessments. Assessments were completed either online or via phone.

#### Use of Other Treatment Programs

Non-assigned treatment use was measured by two items on the 3-month follow-up assessment. The first item asked: Which of the following products or methods have you tried in the last 3 months? (check all that apply). Answer options included treatment methods assigned in the QSN intervention condition but not in the Active Lives control condition (nicotine gum, nicotine patch, nicotine lozenge, nicotine spray, nicotine inhaler, other nicotine replacement product, Zyban), treatment methods that were not assigned in either the treatment or control condition (group cessation program or class, individual counseling [including by telephone], hypnosis or acupuncture, pamphlets or books), or none of the above. A separate item asked: Have you used any other Internet smoking cessation programs since first using the QSN/Active Lives program?

We created two composite measures of non-assigned treatment usage: one measure was defined as the sum of non-assigned treatments reportedly used (score ranged from 0 - 5; treatments included individual counseling, group counseling, hypnotherapy/acupuncture, other Web programs, and pamphlets/books), and the other composite was defined as the yes/no dichotomy describing whether any of these non-assigned treatments had been used.

#### Use of Pharmacotherapy Products

As noted above, participants were asked (yes/no) whether they had used any pharmacotherapy products (nicotine gum, patch, lozenge, spray, and inhaler) or Zyban^®^ since the start of their involvement in the SHIP study. Use of NRT products was explained and strongly recommended in the QSN condition, but the topic was purposefully omitted in the Active Lives control condition. We created two composite measures of using pharmacotherapy: one measure was defined as the sum of non-assigned treatments reportedly used (nicotine gum, patch, lozenge, spray, inhaler, and Zyban^®^), and the score ranged from 0 - 6; and the other measure was defined as the yes/no dichotomy describing whether any of these pharmacotherapy programs had been used.

#### Participant Exposure

The extent to which participants accessed their assigned Web-based program was measured unobtrusively using a combination of database tracking and Web-server log analysis [14] to determine both number and duration of visits (sessions). A composite measure of participant exposure was defined as the mean of standard scores for the number of visits and total time spent across all visits.

#### Smoking Cessation Outcomes

Participant 7-day point prevalence smoking abstinence was assessed both at 3 and 6 months by asking: Have you smoked any cigarettes in the last week, even a puff? The more rigorous repeated point prevalence of self-reported smoking cessation at both the 3- and 6-month assessments was also used. As with other Web-based programs and large-scale self-help interventions for tobacco cessation (eg, [15,16,17,18,19]), we did not collect biochemical measures to verify self-reported tobacco abstinence. Outcomes are reported using both Intent-to-Treat (ITT) analyses (missing cases imputed as smokers) and complete case analyses (based only on cases that completed assessments).

We also measured putative predictors of smoking cessation. Baseline assessment included an item about friends’ smoking (Most of my friends and acquaintances smoke [1 = Not true of me at all, 7 = Extremely true of me]), two items on nicotine dependence (I usually want to smoke right after I wake up [1 = Not true of me at all, 7 = Extremely true of me]; How strong are your urges when you first wake up in the morning? [1 = Not strong at all, 7 = Extremely strong]), and five self-efficacy items. The self-efficacy items all used the same 7-point rating scale (1 = Not at all confident, 7 = Very confident), and they included a global item (If you decided to quit smoking, how confident are you that you could quit) and four items that asked about specific settings/circumstances (How confident are you that you can resist smoking when you are feeling bored or restless?; How confident are you that you can resist smoking when you are angry, frustrated, or tense?; How confident are you that you can resist smoking when you drink alcohol?; How confident are you that you can resist smoking when you are around others who are using it?).

### Statistical Analyses

Logistic and standard regression tests were used to test the relation between participant characteristics and reported use of non-assigned treatments. Similar analyses were used to test the relation of non-assigned treatment use, controlling for treatment condition, on point prevalence smoking cessation at 3 months, at 6 months, and for repeated point prevalence that considered smoking status at both 3- and 6-month follow-up assessments.

## Results

### Assessment Completion and Participant Attrition

Consistent with many Web-based tobacco cessation interventions, the SHIP trial experienced significant attrition over the follow-up interval. Of the 2318 participants initially randomized, 44.3% (N = 1028) completed the 3-month assessment, 32.8% (N = 909) completed the 6-month assessment, and 27.2% (N = 631) completed both assessments. No between-group differences in attrition were found.

**Figure 1:**
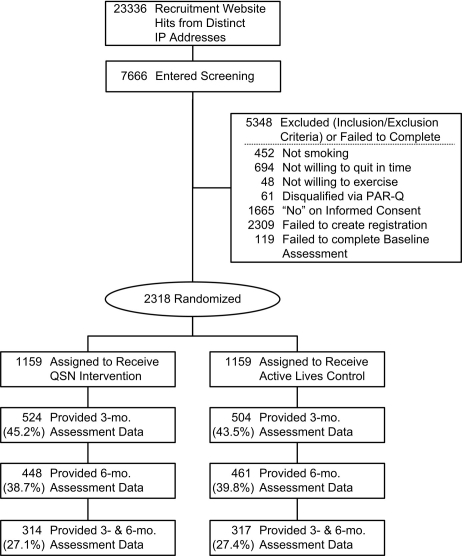
CONSORT diagram for SHIP RCT

### Use of Non-assigned Treatments

A total of 24.1% (248/1028) of participants reported that they had used some other smoking cessation program during the first 3 months they were enrolled in the SHIP trial. The types of non-assigned treatments used depicted in Table 1 show that a small proportion of participants used group counseling (2.3%) and individual counseling (1.7%), and substantially more participants reported using hypnotherapy/acupuncture (4.5%). More striking was the reported use of pamphlets/books (12.6%) and other Web-based smoking cessation programs (9.0%). Differences between the QSN and Active Lives conditions were not significant in terms of the number of non-assigned treatments used (Mean = 0.29, SD = 0.60 vs Mean = 0.31, SD = 0.59; unequal variance *t*_1025.5_ = 0.63, *P =* .530) or in terms of any non-assigned treatment use: 23.1% (121/524) vs 25.2% (127/504); χ^2 ^(1, N = 1028) = 0.62, *P* = .43). As a result, we hereafter describe non-assigned treatment usage patterns for the total sample of participants (collapsed across condition) for whom assessment data were available.

**Table 1:** Participant use of non-assigned treatments: reported at the 3-month follow-up

	QSN Intervention	Active Lives Control	Total
Non-assigned treatment	N = 524	N = 504	N = 1028
Individual counseling	7 (1.3%)	10 (2.0%)	17 (1.7%)
Group counseling	15 (2.9%)	9 (1.8%)	24 (2.3%)
Hypnotherapy/acupuncture	22 (4.2%)	24 (4.8%)	46 (4.5%)
Other Web-based programs	43 (8.2%)	50 (9.9%)	93 (9.0%)
Pamphlets/books	65 (12.4%)	65 (12.9%)	130 (12.6%)

The QSN intervention condition explained and recommended the use of pharmacotherapy products whereas the Active Lives control condition did not. As can be seen in Table 2, the two conditions did not differ in terms of the number of pharmacotherapy products used as reported at the 3-month assessment (QSN: Mean = 0.68, SD = 0.86; Active Lives: Mean = 0.60, SD = 0.83; unequal variance *t*_1026.97_ = -1.54, *P =* .062). However, significantly more participants in the QSN condition were found to have used at least one pharmacotherapy product: 50.0% (262/524) vs 43.8% (221/504); χ^2 ^(1, N = 1028) = 3.90, *P* = .048. Participants made greatest use of nicotine patches and Zyban^®^. The use of pharmacotherapy and non-assigned treatments types showed a small but marginally significant correlation: *r*_1028_ = .061, *P* = .05.

**Table 2:** Participant use of pharmacotherapy products: reported at the 3-month follow-up

	QSN Intervention	Active Lives Control	Total
	N = 524	N = 504	N = 1028
Nicotine gum	65 (12.4%)	65 (12.9%)	130 (12.6%)
Nicotine patch	143 (27.3%)	124 (24.6%)	267 (26.0%)
Nicotine lozenge	40 (7.6%)	25 (5.0%)	65 (6.3%)
Nicotine spray	5 (1.0%)	5 (1.0%)	10 (1.0%)
Nicotine inhaler	22 (4.2%)	14 (2.8%)	36 (3.5%)
Zyban^®^	71 (13.5%)	56 (11.1%)	127 (12.4%)

#### Non-assigned Treatment Use and Participant Characteristics

Each of six participant baseline characteristics (age, gender, marital status, education, rurality, cigarettes smoked/day) was tested using univariate logistic regression for its relation to any non-assigned treatment use. Non-assigned treatment use (composite dichotomous yes/no measure) was found to be positively related to being female (OR = 1.90, 95% CI = 1.34 - 2.69, *P* < .001) but negatively related to lower levels of education (no high school degree: OR = 0.38, CI = 0.16 - 0.88, *P* < .023; high school graduate: OR = 0.53, CI = 0.36 - 0.78, *P* = .001). The same findings obtained when we tested gender and education together using a multivariate logistic regression.

#### Non-assigned Treatment Use and Participant Exposure

A Pearson correlation was used to test the relation between participant exposure and the number of non-assigned treatment types used. The result indicated little relation between participant exposure to the assigned Web-based program and the use of non-assigned treatments: *r*_1028_ = .059, *P* = .06.

#### Non-assigned Treatment Use and Smoking Cessation

A total of 202 participants reported not smoking at 3 months: 19.6% complete case (202/1028) and 8.7% ITT (202/2318). At the 6-month assessment, 232 participants reported not smoking: 25.5% complete case (232/909) and 10.0% ITT (232/2318). A total of 89 participants who completed both the 3- and 6-month follow-up assessments indicated that they were not smoking on each occasion: 14.1% complete case (89/631) and 3.8% ITT (89/2318). No statistically significant between-group differences in smoking cessation were found at these assessment points [11].

We used univariate logistic regression to determine the relation of each of the five types of non-assigned treatment use and smoking cessation at 3 months, at 6 months, and the 3- and 6-month repeated point prevalence measure. Only use of other Web programs was found to be related to smoking cessation: it was positively related at the 3-month assessment (OR = 2.63, CI = 1.67 - 4.14, *P* < .001), at the combined 3- and 6-month assessments (OR = 2.09, CI = 1.11 - 3.91, *P* = .022), but not at the 6-month assessment (OR = 1.63, CI = .946 - 2.79, *P* = .079). The significant effect of using other Web programs on smoking cessation obtained even when gender was included in a multivariate logistic regression. The composite measure (sum of non-assigned treatment types used) was found to be unrelated to smoking cessation outcomes.

In addition, a test for the moderator effect of condition and non-assigned treatment usage on smoking cessation failed to find any noteworthy interaction effects at either the 3- or the 6-month outcome. Indeed, when we eliminated from the analysis data of participants who indicated that they had used non-assigned treatments, no effect for condition on smoking cessation outcome emerged at 3 months, 6 months, or the combined 3- and 6-month assessments.

Univariate logistic regression revealed four putative predictors of smoking cessation to be significantly related to non-assigned treatment use: self-efficacy to quit when using alcohol (OR = 1.09, CI = 1.00 - 1.18, *P =* .040), most friends and acquaintances smoke (OR = 0.91, CI = 0.85 - 0.97, *P =* .005), urges to smoke upon awaking (OR = 1.14, CI = 1.06 - 1.23, *P* < .001), and smoking upon awaking (OR = 1.15, CI = 1.07 - 1.25, *P* < .001). Since the two dependence items were highly correlated (*r*_1028_ = .770, *P* < .001), we included only the item that asked about smoking upon awaking with the other two variables in a multivariate logistic regression which essentially confirmed the univariate results just described.

#### Pharmacotherapy Use and Smoking Cessation

Univariate logistic regression revealed that the sum of pharmacotherapy products reported at 3 months used was unrelated to smoking cessation at 3 months (OR = 1.07, CI = .89 - 1.27, *P =* .475), but the dichotomous measure of any pharmacotherapy product use at 3 months was related to 3-month smoking cessation (OR = 1.42, CI = 1.04 - 1.93, *P =* .027). A similar pattern emerged when we considered smoking cessation at 6 months: the sum of pharmacotherapy products reported at 3 months was not related to 6-month smoking cessation (OR = 0.94, CI = .81 - 1.21, *P* = .944), but the dichotomous measure of any pharmacotherapy product use at 3 months was unrelated to 6-month smoking cessation (OR = 1.41, CI = .99 - 2.01, *P =* .059).

## Discussion

### Strengths & Limitations

Strengths of the current research include the successful use of online marketing strategies to recruit a large sample of 2318 participants and our use of a RCT methodology. Limitations include noteworthy participant attrition—an outcome that has been reported in other Web-based tobacco cessation studies [1,20]. Another possible limitation is the large proportion of women participants: 70.5% (1634/2318) of the full randomized sample in the SHIP RCT and 71.6% (736/1028) of the participants completing the 3-month assessment. Results from the current study indicated that a significantly greater proportion of women than men reported that they used non-assigned treatments. However, gender did not influence the positive relation we found between using non-assigned Web programs and smoking cessation outcomes. Future research is needed to explore in more detail the role of gender on the prevalence and helpfulness of using non-assigned treatments.

Additional debriefing questions were not included in the assessment that could have helped to illuminate reasons for using non-assigned treatments. For example, questions could have probed participants’ attitudes about, and reasons for, using other smoking cessation programs, and the extent that they thought non-assigned treatments were helpful and personally relevant. It would be interesting to know whether study participants felt that outside programs were relatively more or less helpful than the treatment methods that were assigned. In addition, we could have asked more specifically about the timing of when participants used non-assigned treatments.

### Conclusions

The incidence of using non-assigned treatments is quite difficult to gauge given that most publications fail to report upon this phenomenon. An exception is Strecher and colleagues [16] who reported that 32.6% (461/1415) of participants in a Web-based smoking cessation trial reportedly used non-assigned smoking cessation programs or aids during the treatment and follow-up period. The use of non-assigned treatments will probably be related to the extent to which treatment options are well-publicized, thought to be effective, and readily available to use. In our study, the number of pharmacotherapy products used was equivalent in the two conditions, even though this use was explicitly emphasized in QSN and purposefully ignored in the Active Lives control. The observed high levels of pharmacotherapy in our control condition is consistent with population data showing that 32.2% of 29,537 US smokers surveyed indicated that they used medication to help them try to quit smoking in the past year [21].

The phenomenon of using non-assigned treatments may be particularly likely among participants of Web-based RCTs who demonstrated their Web foraging skills [22] when they were recruited online. Finding other credible and attractive online behavior-change resources and programs requires minimal work and effort. The use of non-assigned treatments may also be more likely during extended follow-up periods, when participants who have been unsuccessful in changing their behavior, but who remain motivated, may decide against waiting to complete a final follow-up assessment before they begin to explore new treatment options.

The frequency and timing of asking participants about their use of non-assigned treatments deserves careful consideration. Because of the substantial attrition found in many Web-based intervention trials [1], it would be helpful to ask participants about non-assigned treatment use in early assessments. This would make it possible to obtain data from more participants, and it could permit analysis of the possible role of non-assigned treatment use on attrition. Asking about non-assigned treatment use on multiple occasions during follow-up would permit a test of whether non-assigned treatment use mediated treatment outcomes. However, questioning participants about their use of non-assigned treatments could also have the significant—and potentially undesirable—reactive effect of encouraging participants to engage in non-assigned treatment use. We recommend that Web-based interventions should routinely debrief participants about their use of non-assigned treatments as part of the final follow-up assessment. Asking at earlier points in the assessment phase warrants careful scrutiny to determine the extent to which such questioning might be reactive.

It is impractical to require Web-based RCT participants to refrain from using alternative treatment programs or to avoid treatment-seeking from other sources. We recommend that the use of non-assigned treatments should not be grounds for participant exclusion from Web-based behavior change interventions. Instead, Web-based interventions should be evaluated as being part of a larger fabric of ongoing self-help and personal improvement programs that people engage in to accomplish important personal behavioral changes. Before they become study participants—and possibly during the time that they are study participants—individuals are likely to be seeking out available resources, including those readily available on the Internet, some of which they may use in making a serious attempt to change their behavior, as in trying to quit smoking [4,5]. Only through asking participants about non-assigned treatments they may have used and/or treatments they may have sought (eg, [6,23]) will it be possible to determine whether such activities might have a positive effect on achieving goals (as in the use of other Web-based smoking cessation programs) or have a more negative relation with outcome (as in the use of pamphlets/books in the current study).

Research may show that it is beneficial to encourage participants to use other treatment resources to complement what they learn about in the behavior change program presented in their RCT. However, engaging in multiple concurrent treatments—some of which might be contradictory—could be counterproductive [1]. A caution about not trying to do too much at one time seems prudent until research highlights beneficial combinations of treatments and/or it identifies treatment combinations that are contraindicated.

## Abbreviations

ITT: intent-to-treat
NRT: nicotine replacement therapy
PAR-Q: physical activity readiness questionnaire
QSN: quit smoking network
RCT: randomized controlled trial
SHIP: smokers’ health improvement program
